# The spectral sensitivity of *Drosophila* photoreceptors

**DOI:** 10.1038/s41598-020-74742-1

**Published:** 2020-10-26

**Authors:** Camilla R. Sharkey, Jorge Blanco, Maya M. Leibowitz, Daniel Pinto-Benito, Trevor J. Wardill

**Affiliations:** 1 Department of Ecology, Evolution and Behavior, University of Minnesota, Saint Paul, MN 55108 USA; 2 Department of Physiology, Development and Neuroscience, University of Cambridge, Cambridge, CB2 3EG UK

**Keywords:** Colour vision, Cell biology, Visual system

## Abstract

*Drosophila melanogaster* has long been a popular model insect species, due in large part to the availability of genetic tools and is fast becoming the model for insect colour vision. Key to understanding colour reception in *Drosophila* is in-depth knowledge of spectral inputs and downstream neural processing. While recent studies have sparked renewed interest in colour processing in *Drosophila*, photoreceptor spectral sensitivity measurements have yet to be carried out in vivo. We have fully characterised the spectral input to the motion and colour vision pathways, and directly measured the effects of spectral modulating factors, screening pigment density and carotenoid-based ocular pigments. All receptor sensitivities had significant shifts in spectral sensitivity compared to previous measurements. Notably, the spectral range of the Rh6 visual pigment is substantially broadened and its peak sensitivity is shifted by 92 nm from 508 to 600 nm. We show that this deviation can be explained by transmission of long wavelengths through the red screening pigment and by the presence of the blue-absorbing filter in the R7y receptors. Further, we tested direct interactions between inner and outer photoreceptors using selective recovery of activity in photoreceptor pairs.

## Introduction

Colour cues in the natural environment are used by many insects, guiding behaviours such as prey detection, mate selection and more general tasks such as flight navigation. To detect and distinguish wavelength cues, the output of two or more photoreceptors with different spectral sensitivities must be compared. This colour-opponency system has been well explored in vertebrates but only more recently has insect colour opponency been characterised, using *Drosophila* as the model^1,2^. *Drosophila melanogaster* is fast becoming the model for insect colour vision due to the wide range of genetic tools available. Surprisingly, despite new advances in our understanding of more complex motion and colour visual processing in *Drosophila,* the characterisation of photoreceptor spectral sensitivity in this species has not been investigated since it was first revealed 20 years ago^3,4^.

**Figure 1:**
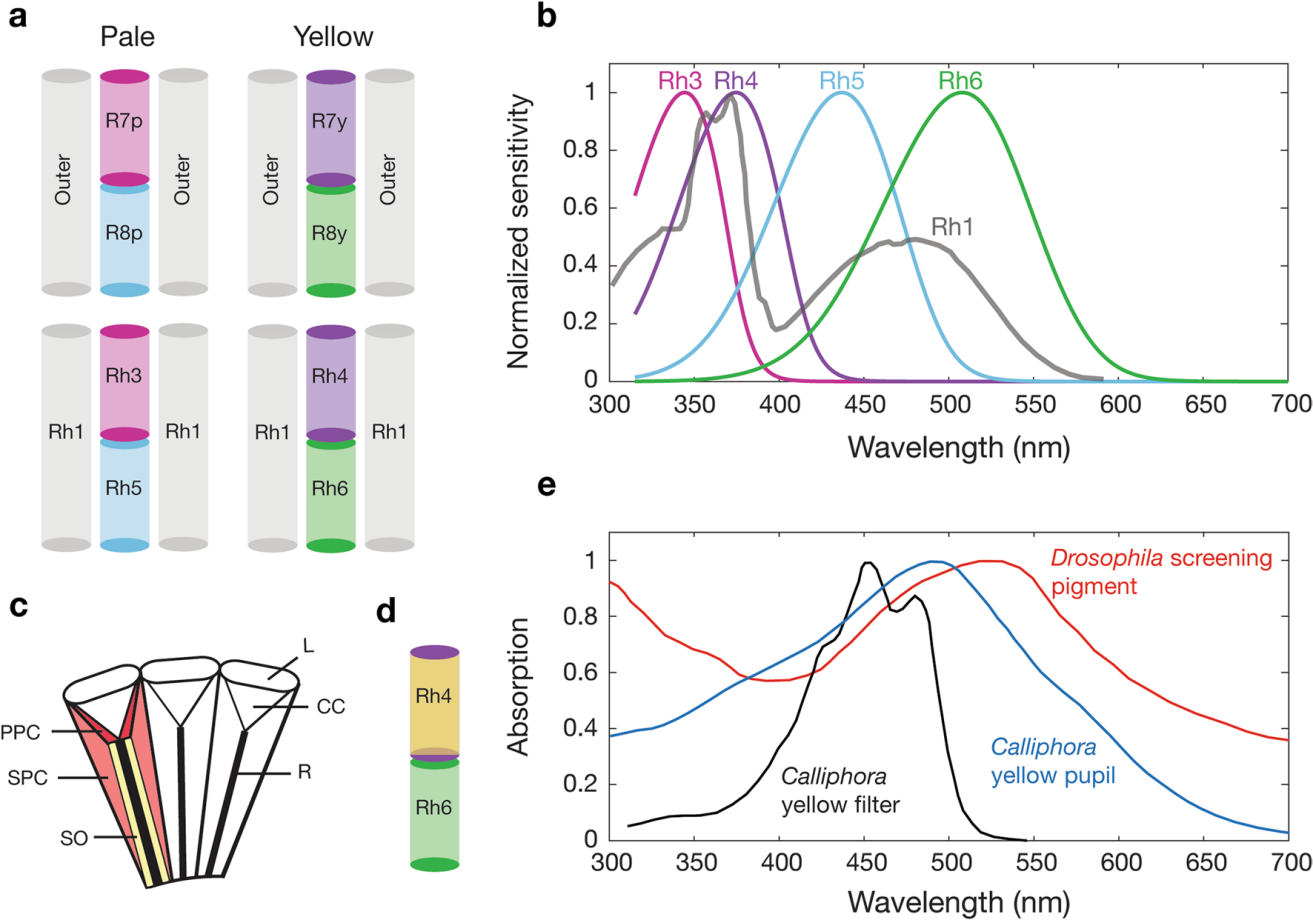
An overview of *Drosophila* photoreceptors, visual pigments and fly ocular pigments. (**a**) The arrangement of inner R7/R8 and outer receptors in pale- and yellow-type ommatidia of *Drosophila* and the opsins expressed in each. (**b**) Spectral sensitivities of visual pigments Rh3–6 modelled using visual pigment templates and previous sensitivity estimates^4^. Rh1 (redrawn^4^) has a characteristic shape owing to a blue-green sensitive visual pigment coupled to a UV-sensitising pigment. (**c**) Longitudinal section diagram of an insect compound eye indicating the distribution of screening pigment in primary pigment cells (PPC) and secondary pigment cells (SPC) that optically isolate the corneal lens (L), crystalline cone (CC) and rhabdom (R). The soma (SO) of the photoreceptors contain mobile pigment granules that form the fly pupil. (**d**) Location of the blue-absorbing yellow filter alongside opsin Rh4 in the R7y rhabdom. (**e**) Absorption of red *Drosophila* screening pigment, *Calliphora* yellow pupillary pigment and the blue-absorbing yellow filter (redrawn^20,21,22^, original source for pupil data^39^).

The *Drosophila* compound eye is formed from approximately 800 ommatidial units, each comprising 6 outer (R1–6) and 2 inner photoreceptors (R7 and R8) with an open rhabdom structure (Fig. 1a). Sensitivity of the photoreceptors is largely determined by the underlying visual pigment and ommatidia can be subdivided into two major classes, ‘pale’ (p) or ‘yellow’ (y), owing to the appearance of the inner pair of photoreceptors in transmitted light, with the latter possessing a blue-absorbing yellow filter in the R7y receptor alongside the UV-sensitive Rh4 visual pigment^5^. In the outer receptors of all ommatidia, opsin Rh1 confers broadband blue-green sensitivity (λ_max_ = 478 nm) with an additional peak in the UV due to an associated carotenoid-derived sensitising pigment^4,6^. Inner receptor R7 cells express UV-sensitive Rh3 (R7p) or Rh4 (R7y) and the proximal receptor R8 cells express either blue-sensitive Rh5 (R8p) or green-sensitive Rh6 (R8y)^3,4^ (Fig. 1a). Direct intracellular recordings of the inner photoreceptors have not been possible due to their small size and stochastic distribution across the retina. Instead, peak sensitivities for Rh3–Rh6 have been estimated from microspectrophotometery (MSP), visual pigment extracts and electroretinography (ERG) with ectopic expression of inner receptor opsin in the more numerous outer receptors, using white-eyed *Drosophila*^3,4^. Although this enabled the underlying visual pigment sensitivities to be measured (Rh3–Rh6 λ_max_: 345, 375, 437, 508 nm; Fig. 1b), these studies were unable to quantify the sensitivity of each when measured in vivo*,* in the photoreceptor cells where the opsins are normally expressed along with their naturally associated ocular screening pigment and photoreceptor filtering pigments (Fig. 1c–e).

The spectral sensitivity of a cell is not dictated solely by the underlying visual pigment but is shaped also by numerous optical filters, including screening pigment that optically isolates neighbouring ommatidia (Fig. 1c). In the *Drosophila* compound eye, the brown ommochrome screening pigment, xanthommatin is present in both primary and secondary pigment cells. Red drosopterin is also present in the secondary pigment cells, which gives *Drosophila* its characteristic red eye colour^7,8^ A yellow pigment present in the soma of the photoreceptors^9^ modulates light input to the rhabdom^10^ and endows the photoreceptors with a pupillary mechanism. In addition to this, dipteran flies have carotenoid-based pigments: a sensitising pigment that contributes additional sensitivity in the UV through energy transfer to the visual pigment^6^ (Rh1; Fig. 1c) and a blue-absorbing yellow filter in the R7y cells^5^ (Fig. 1d,e). *Drosophila* screening pigment absorbance peaks at 520 nm and becomes more transmissive at longer wavelengths (Fig. 1e), an adaptation thought to maximize the reconversion of Rh1 rhodopsin from its metarhodopsin, which peaks at 566 nm^4^. Early studies pointed towards a red receptor in dipterans^11^, later shown to be an artefact of long wavelength light leakage^12^. It has been argued that this red sensitivity may be of little consequence to the visual system of such flies when under ecologically-relevant levels of illumination^12^. Furthermore, it is thought that the wavelength peak of *Drosophila* Rh6 (508 nm) is short-wavelength shifted when compared to flies with brown screening pigment (e.g. *Musca,* 520 nm), as an adaptation to reduce the absorption of red light. The effect of light leakage on the *Drosophila* Rh6 visual pigment has yet to be tested in vivo.

Much of what is understood about animal colour vision is derived from studies of vertebrates and only recently have investigations revealed the neural basis of colour information processing in insects. Colour information processing in *Drosophila* has been investigated using behavioural experiments^13–15^, modelling^16^ and by visualising neural responses directly in the fly brain, in response to light stimulation with genetic manipulation^1,2^. Colour opponency begins at the first visual synapse, the photoreceptor terminal, with reciprocal inhibition occurring between paired R7 and R8 receptors^1^. Additionally, there is transfer of information between inner and outer receptors via gap junctions in the lamina that is thought to enhance the sensitivity of the motion detection pathway^17^. Direct opponency between photoreceptors by way of opponent voltage responses have been demonstrated to occur in other insects and is well characterised in some lepidopteran species^18,19^. Such opponency in the photoreceptor soma was not detected in *Drosophila* in a recent study that inferred photoreceptor response from axonal measurements, using genetic dissection and two-photon calcium imaging^2^. In this study, we aimed to further test for potential interactions at the level of the photoreceptor soma by directly measuring photoreceptor voltage responses, with activity of photoreceptor pairs restored.

Here we report the first complete in vivo characterisation of *Drosophila* spectral sensitivity, for each receptor type, by selectively restoring photoreceptor activity in flies with no receptor activity (*norpA*) and testing response using ERG. We characterise the sensitivity of photoreceptors in flies with screening from distal receptors and ocular pigments intact and we test the effect of screening pigment and the blue-absorbing yellow filter on inner receptors. We reveal significant shifts in spectral sensitivity for all receptor sensitivities and a large 92 nm shift in sensitivity of the Rh6 visual pigment when measured in its native photoreceptor (R8y) from 508 to 600 nm. We also find that the blue-absorbing yellow filter refines sensitivity of the Rh4 visual pigment in R7y photoreceptors. Furthermore, we explore the effect of reciprocal inhibition between inner photoreceptors on spectral response at the level of the photoreceptors and to what extent the outer photoreceptors input to this system. Our results indicate that the input from inner photoreceptors to downstream neuronal process is linear and provide no clear evidence for direct interactions at the photoreceptor level. Spectral modulation can be seen however between inner and outer receptors, providing further evidence of possible interactions between motion and spectral channels in the lamina.

## Results

### Single opsin rescues, Rh1, Rh3, Rh4, and Rh5

**Figure 2:**
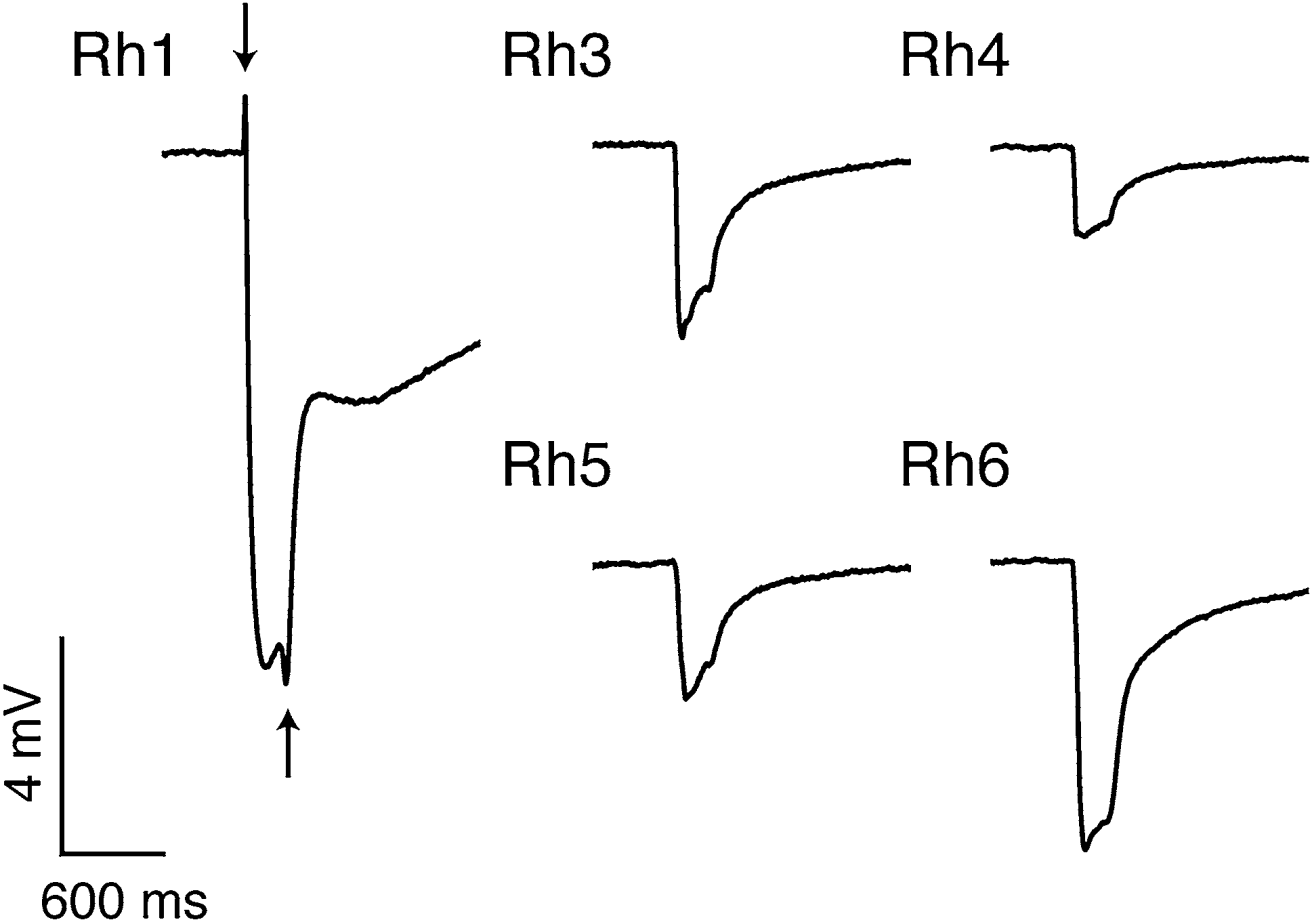
Example ERG traces of single opsin rescue flies in response to a 200 ms pulse of light at photoreceptor saturation (3.60 × 10^16^ photons cm^−2^ s^−1^). Arrows indicate on and off transients in the outer receptors, the postsynaptic potentials in second-order neurons.

**Figure 3:**
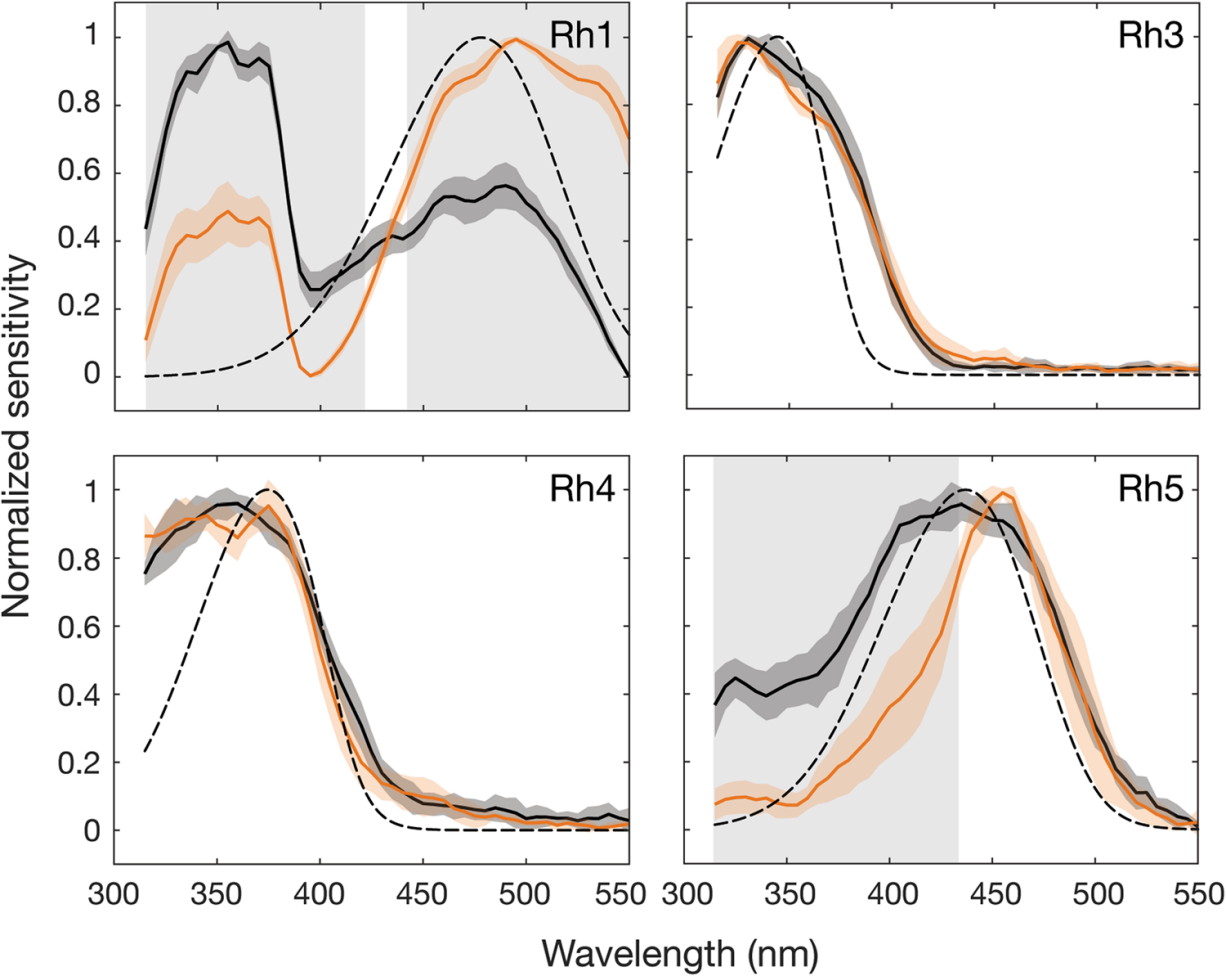
Spectral sensitivity of red and orange eye flies with selectively rescued photoreceptor responses. Normalized spectral sensitivity of red eye (black lines) and orange eye (orange lines) flies with rescued activity of receptors with Rh1, Rh3, Rh4 or Rh5. Modelled visual pigment templates (dashed lines), based on previous estimates^4,40^. Error shown is standard deviation. Shading denotes significance between red and orange eye flies using a two-sample Student’s t-test at *p* ≤ 0.001. For V-log(I) curves, (see Supplementary Figure S1). n = 6 for all spectral sensitivity tests. For statistical test results see Supplementary Data.

We generated flies with rescued activity of specific photoreceptor types (single opsin rescue flies) to test the spectral sensitivity of the inner R7p (Rh3), R8p (Rh5), R7y (Rh4), R8y (Rh6) and outer receptors (Rh1), using ERG. We were able to measure reliably from all single opsin rescue genotypes. Rh1 rescue flies exhibited characteristic on and off transients at the start and end of the 200 ms light pulse, absent in Rh3–Rh6 rescue flies (Fig. 2). The spectral responses of rescue flies Rh3, Rh4 and Rh5 with wild-type screening pigment had significant shifts in spectral sensitivity for portions of their detection range compared with previous characterisations (Fig. 3, black dashed traces). Sensitivities of Rh3 and Rh4 rescue flies peaked at 330 and 355 nm, respectively, which is significantly short wave shifted from estimates of peak sensitivity with ectopic expression of Rh3 and Rh4 in outer receptors of white eye flies (334 and 375 nm^3^). However, they were in good agreement with maximal sensitivities derived from visual pigment extracts (331 and 355 nm^4^).

The peak sensitivity of the Rh5 rescue flies (435 nm) was similar compared to previous measurements of the visual pigment (437 nm)^4^ but the response we measured had significantly boosted sensitivity in the ultraviolet range. The spectral sensitivity response of R1–R6 (Rh1) was also significantly altered from a simple visual pigment template. There were notably three fluctuations in the waveform around the visual pigment peak (Fig. 3). The spectral profile of Rh1 rescue flies also shows the characteristic triple-peaked UV spectrum of the UV-sensitising pigment coupled with the curve of the visual pigment peaking at 490 nm (Fig. 3). The fine structure of the peaks however is less evident in this study than in other published spectra, due to smoothing of the data and the resolution of spectral sensitivity tests, at 5 nm steps between wavelengths. Spectral curves from all four photoreceptor cell types were broader than predicted by a visual pigment template.

To test the effect of screening pigment on photoreceptor response, we examined opsin rescue flies with wild-type (red-eye) and reduced screening pigment (orange-eye). The spectral sensitivities of Rh3 and Rh4 single opsin rescue flies were not affected by a reduction of screening pigment. However, a reduction in screening pigment led to narrowing of the Rh5 rescue response (Fig. 3, orange traces) and a bathochromic (long-wave) shift in the peak sensitivity by 20 nm, from 435 to 455 nm. Screening pigment reduction in Rh1 rescue flies caused an increase in sensitivity at the visual pigment peak, reducing the relative sensitivity in the UV region.

**Figure 4:**
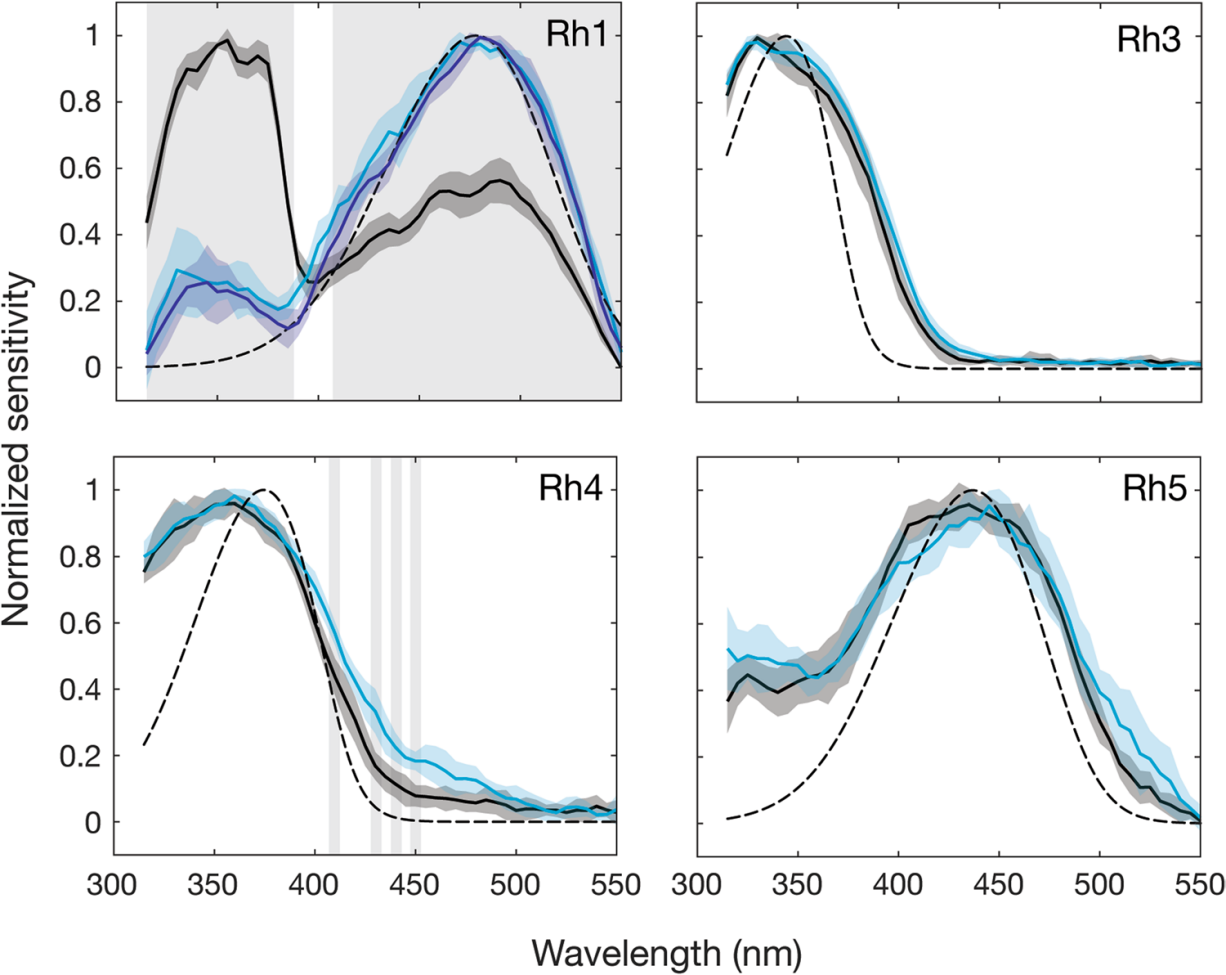
Spectral sensitivity of flies with carotenoid deprivation and selectively rescued photoreceptor responses. Normalized spectral sensitivity of red-eye flies raised on a regular diet of yellow cornmeal (black) or carotenoid-deprived flies raised on yeast-glucose for one (light blue) or two (dark blue) generations, in the case of Rh1 flies. Modelled visual pigment templates (dashed lines) based on previous estimates^4,40^. Error shown is standard deviation. Shading denotes significance between normal and carotenoid-deprived (one generation) red-eye flies using a two-sample Student’s t-test at *p* ≤ 0.001. n = 6 for all spectral sensitivity tests. For statistical test results see Supplementary Data.

Carotenoids were removed from the diet of red eye rescue flies to test for the presence of carotenoid pigments in the outer receptors (Rh1) and inner R7p (Rh3), R7y (Rh4) and R8p (Rh5) receptors. The response of the UV-sensitising pigment coupled to visual pigment Rh1 was effectively removed by carotenoid deprivation after one generation on yeast-glucose food and showed no further change in spectral shape after two generations (Fig. 4). Carotenoid deprivation had no effect on the spectral response of Rh3 opsin rescue flies but broadened the response in Rh4 rescue flies above 400 nm (Fig. 4). Responses of Rh5 rescue flies were reduced by carotenoid deprivation (Supplementary Figure S1) but the profile of the sensitivity curve was unchanged (Fig. 4).

### Single opsin rescue Rh6

**Figure 5:**
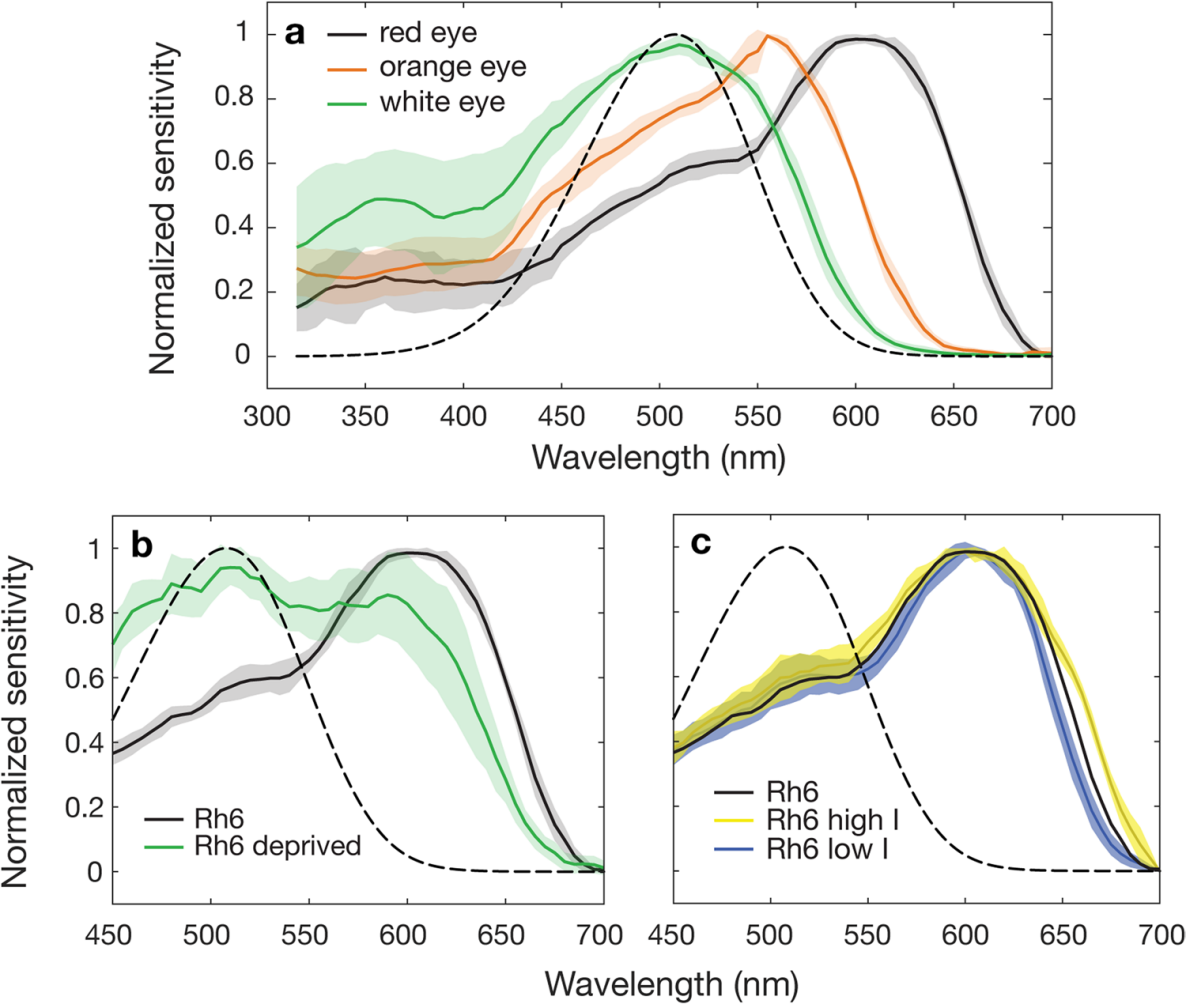
The effect of screening pigment, carotenoid deprivation and ectopic expression on the spectral sensitivity of the Rh6 visual pigment. (**a**) Normalized spectral sensitivity of Rh6 rescue flies with red (black solid), orange (orange) or white eyes with Rh6 expressed in outer receptors (green), peaking at 600, 555 and 510 nm, respectively. Spectral sensitivity template of the Rh6 visual pigment peaking at 508 nm (dashed line). (**b**) Normalized spectral sensitivity of Rh6 rescue flies with red eyes raised on a regular (black solid line) or carotenoid-free diet for one generation (green line). (**c**) Response curves of Rh6 rescue flies tested at a high and low intensity that differed by 1.05 log units of light, 0.45 below and 0.6 above the intensity at half maximum response. All error shown is standard deviation. n = 6 for all spectral sensitivity tests. The same six Rh6 red-eye rescue flies were used for (**a**—red eye) and (**c**—Rh6 low intensity and Rh6 high intensity).

In Rh6 single opsin rescue flies, where R8y activity was rescued, the spectral response was far broader than expected and considerably shifted in peak wavelength sensitivity from the previous estimate (508 nm) to 600 nm when the sensitivity was measured in vivo (Fig. 5a). This large bathochromic shift was reversed by 45 to 555 nm by the reduction of screening pigment in orange-eye flies. Sensitivity of white-eye mutants where screening pigment was absent and Rh6 was expressed in the outer receptors peaked close to the predicted peak wavelength of the visual pigment (508 nm), at 510 nm (Fig. 5a).

We aimed to test the effect of the carotenoid-based blue-absorbing yellow pigment on the sensitivity of Rh6 by means of carotenoid deprivation. When the yellow pigment was removed, two peaks of sensitivity could be seen, one at the peak sensitivity of Rh6 (508 nm) and the other close to the peak of the flies raised on regular carotenoid rich diet (600 nm) (Fig. 5b). There was no change in the shape of the spectral response between one and two generations of carotenoid deprivation (Supplementary Figure S2). As carotenoid deprivation reduces the overall sensitivity of the photoreceptor by chromophore depletion, flies must be tested at a higher light intensity. As such, due to the limit of light in the system, flies were tested at the lower end of the stimulus–response V-log(I) curve (Supplementary Figure S1). To simulate these potential confounding conditions, we tested Rh6 rescue flies raised on a regular diet, across 1.05 log units of light, 0.45 below and 0.6 above the normal testing intensity. This range allowed testing of decreased and increased intensity, while remaining in the linear portion of the V-log(I) curve (Supplementary Figure S1). Spectral responses were relatively unchanged by light intensity with minor broadening and narrowing of the spectral curve occurring at higher and lower light intensities, respectively (Fig. 5c). Importantly however, the spectral shape of carotenoid-deprived Rh6 rescue flies, specifically the peak at 510 nm could not be replicated.

### Double opsin rescues

**Figure 6:**
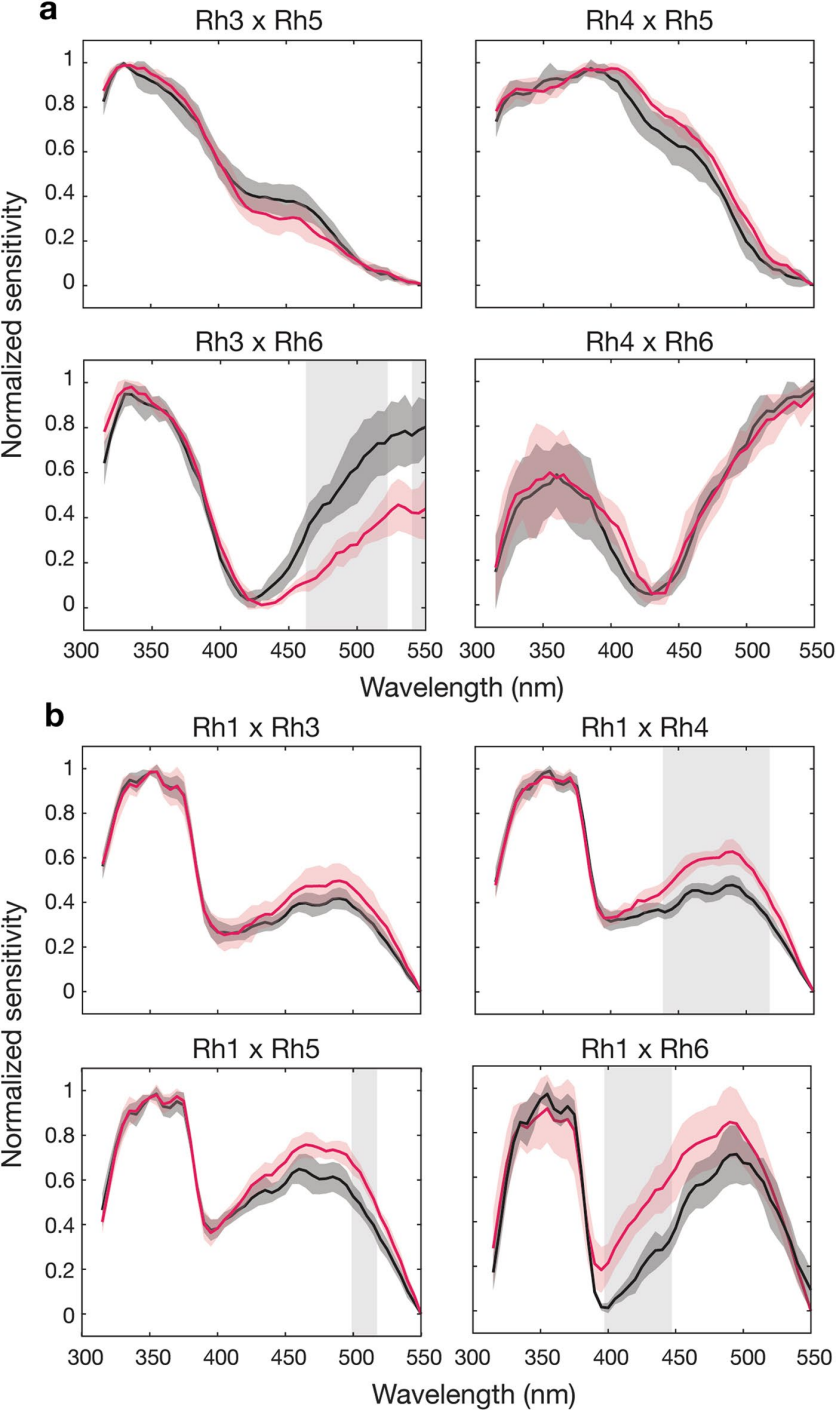
Spectral sensitivity of double opsin rescue flies and the sum of equivalent single rescue responses. (**a**) Normalized responses from double opsin rescue flies with the activity of two photoreceptor types active (pink) compared with the algebraic sum of the single rescue responses (black). Animals were tested at two intensities, derived from the V-log(I) response at Rh3/Rh4 peak sensitivities and Rh5/Rh6 peak sensitivities (not shown, see Supplementary Figure S3). (**b**) Double opsin rescue flies with Rh3–6 and Rh1 (pink) compared to the sum of single rescue flies (black). Double rescue flies were tested at intensities using V-log(I) responses at the Rh1 peak sensitivity and Rh3/Rh4/Rh5/Rh6 peak sensitivities (not shown, see Supplementary Figure S3). n = 6 for all spectral sensitivity curves. All error shown is standard deviation. Shading denotes significance between double rescue response and sum of singles using a two-sample Student’s t-test at p ≤ 0.001. For statistical test results see Supplementary Data.

To test for potential spectral modulation at the photoreceptor level, responses from flies with two active photoreceptor types (double opsin rescue flies) were compared with the sum of corresponding single photoreceptors. We found no difference in spectral shape when double rescue flies were tested at different intensities, according to the two V-log(I) tests carried out at each visual pigment peak sensitivity, with the exception of a single wavelength in the Rh3 and Rh5 double rescue flies (Supplementary Figure S3). If there were interactions between photoreceptors we would expect the sum of the single receptors to differ from that of the double rescue. In R7 and R8 receptor double rescues, photoreceptors were active either within the same ommatidia (e.g. Rh3 and Rh5: R7p and R8p) or in different ommatidia (e.g. Rh4 and Rh5: R7y and R8p). In both cases, highly similar responses were found and these approximately equated to the sum of the single rescues, with the exception of the Rh3 and Rh6 double rescue (Fig. 6a). Although a clear difference in spectral profile can be seen between the expected sum of Rh3 and Rh6 and the corresponding double opsin rescue (Fig. 6a), this difference is no longer present in the non-normalized data (Supplementary Figure S4). The sum of the Rh3 and Rh6 spectral responses do closely match those of the double rescue flies (Rh3 and Rh6) but only when compared to data collected at the intensity calculated from the V-log(I) experiment at the corresponding peak of the opsin in question.

Significant differences in spectral shape between the expected sum of single photoreceptor responses and double opsin rescues between inner and outer receptors were observed for outer receptors in combination with Rh4, Rh5 and Rh6 (Fig. 6b). In all three cases, the observed shift in the sensitivity curve is due to a lower than expected response in the UV, which translates to a relatively higher response at wavelengths higher than 400 nm, upon normalization (Supplementary Figure S4). The sum of all mean responses from each photoreceptor type matches well to the response measured from wild-type flies (Supplementary Figure S5), indicating that the photoreceptor responses do indeed sum linearly when all interactions are combined.

**Figure 7:**
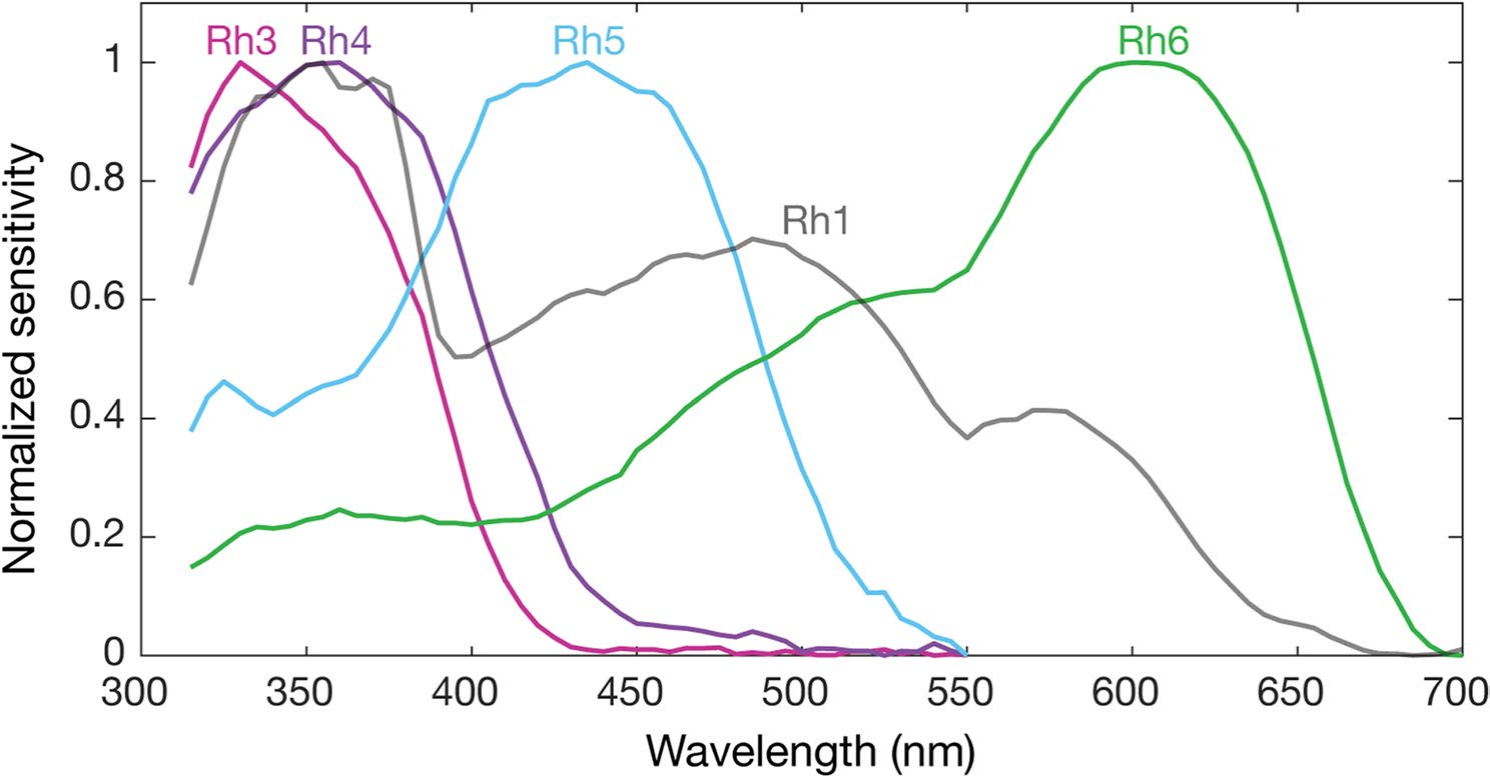
Summary of *Drosophila* photoreceptor sensitivities. (**a**) Normalized mean spectral sensitivity curves of *Drosophila* visual pigments Rh1, Rh3, Rh4, Rh5 and Rh6 measured in red eye flies. n = 6 for each spectral curve. See Supplementary Figure S6 for non-normalized photoreceptor response data.

Finally, we provide a final summary figure that outlines the normalized spectral sensitivity profiles of each photoreceptor and their underlying sensitivities (Fig. 7) and the data for each (see Supplementary Figure S6 and Supplementary Data).

## Discussion

We have shown that using *norpA* flies with activity rescued in selected photoreceptor types alongside ERG enabled the characterisation of spectral sensitivity in *D. melanogaster*, as an alternative to intracellular recordings. The spectral response of the outer receptors, which express Rh1 opsin, is similar in profile and peak sensitivity to previous studies^4^. Three clear fluctuations can be seen between 430 and 490 nm, which may be explained by the presence of an ocular pigment absorbing at approximately 440 and 475 nm, where response dips in the Rh1 spectral curve (Fig. 3). A similar interaction with carotenoids can be seen clearly in the R7y photoreceptors of *Calliphora*, where UV sensitivity is gained by a blue-sensitive visual pigment, coupled to a UV-sensitising pigment and a blue-absorbing yellow filter^20^. The fluctuations we observed may be explained by absorption of off-axis light by the blue-absorbing carotenoid, which in the R7y of *Calliphora* absorbs maximally at approximately 450 nm and 480 nm (Fig. 1e)^20^. In the absence of dietary carotenoids, these fluctuations are no longer evident, further suggestive of an interaction with carotenoid pigments.

When screening pigment was reduced, the sensitivity of the Rh1 visual pigment (λ_max_ = 478 nm) increased relative to the UV-sensitising pigment, indicating that more light is available to stimulate the visual pigment directly in the blue-green region of the spectrum. This may be explained by the reduction in screening of by off-axis light by the blue-green absorbing yellow pupil (Fig. 1e) and by reduced efficiency of UV-transmittance laterally through the eye, compared to longer-wavelength light. This would cause a relatively greater increase in stimulation of the Rh1 visual pigment than UV-sensitising pigment. The decrease in overall sensitivity observed in the reduced screening pigment mutant V-log(I) can be explained by adaptation of the outer photoreceptors in response to background deep red light, used to increase photoconversion of Rh1 metarhodopsin (Supplementary Figure S1).

The sensitivity spectra of inner R7 receptors are similar to their respective visual pigments Rh3 and Rh4^4^ but exhibit broadening. If this broadening were due to self-screening, we might expect to observe narrowing of the spectral curve when the screening pigment is reduced. However, neither R7 receptors were affected by a reduction in screening pigment, suggesting that these spectral curves may be a fair representation of the underlying visual pigment. The spectral profiles of R8 inner receptors are notably modified by the presence of ocular pigments and are poorly described by the underlying visual pigment sensitivities of Rh5 and Rh6^4^. The shift in sensitivity of Rh5 rescue flies when screening pigment levels were reduced is likely due to the increase in off-axis light, which increases direct stimulation of the R8p receptor. The resulting sensitivity curve more closely resembles the underlying sensitivity of the visual pigment^4^. This indicates that the broadening of sensitivity that we observe is likely due to distal screening from the UV-receptor R7p.

The sensitivity curve of Rh6 rescue flies (R8y) with wild-type (red) screening pigment is far broader than expected, with low levels of sensitivity in the UV and a major peak of sensitivity at 600 nm (Fig. 5a). This long-wavelength shift of 92 nm from 508 to 600 nm can be in part explained by the absorption curve of the red screening pigment, which is maximal at 290 and 520 nm^21^ (Fig. 1e). Above 520 nm there is a steady decline in light absorption by the screening pigment. Therefore, above 520 nm, stray light is able to leak through the secondary pigment cells surrounding the R8y receptors and stimulate the long-wavelength tail of the Rh6 visual pigment. This has the effect of increasing the light available to the R8y receptors above 520 nm. This effect can be seen by the hypsochromic (short-wave) shift in peak sensitivity of the reduced screening pigment mutants back towards the peak sensitivity of the Rh6 visual pigment to 555 nm (Fig. 5a). By reducing the effect of the long-wavelength light leakage, the relative absorption of the Rh6 visual pigment is shifted towards its peak sensitivity. We are confident that the absorbance peak of Rh6 in *Drosophila* is indeed close to the previously measured 508 nm as confirmed by our measurements of white eye flies with ectopic expression of Rh6 in the outer receptors (Fig. 5a). We expect that the peak sensitivity of the R8y receptor measured with point-source illumination would match that of the underlying visual pigment (508 nm). However, with wide-field illumination that is perhaps more indicative of the natural light environment, we show that there is a clear effect of light leakage on the sensitivity of the R8y receptor that shifts sensitivity towards the red.

It has been proposed that the Rh1 and Rh6 of *Drosophila* and other red-eyed flies may be sensitive to the longer wavelengths of light that leak through the red screening pigment and as a consequence this would degrade spatial resolution^22^. At high light levels, the movement of pupillary granules within the photoreceptors cause a short-wavelength shift in the peak sensitivity of the outer receptors and increases photoconversion of metarhodopsin^23^. While the Rh1 metarhodopsin (λ_max_ = 566 nm) will readily absorb long wavelength light leaking through the screening pigment, this is not the case for Rh6, whose metarhodopsin absorbs maximally in the blue-green (λ_max_ = 468 nm). The effect of light leakage on the Rh1 visual pigment can be seen as a secondary peak close to 575 nm (Fig. 7). A similar peak has been observed previously in red-eyed *Calliphora* and *Musca* but peaking near 620 nm^11,12^. This likely reflects differences in the absorbance profile of the screening pigment, which in the red-brown eyes of *Calliphora* and *Musca* is long-wavelength shifted^21^. Additionally, screening pigment is packed less densely in *Drosophila* than large flies with red screening pigment^20,22^ and small muscids (e.g. *Coenosia*^24^). In both Rh1 and Rh6 opsin rescue flies, response increases above 550 nm (Fig. 7), coinciding with the increase in transmission of the screening pigment (Fig. 1). This shows that above 550 nm, light leakage through the screening pigment begins to affect photoreceptor response. The relative absorption of the red screening pigment is similar at both 400 and 600 nm, suggesting that the transmission of stray light would be comparable in the two regions of the spectrum. However, while pupillary pigment will absorb stray light at 400 nm, no such mechanism is present that reduces stray long-wavelength light within the photoreceptor. It is not clear to what extent the spatial resolution of R8y photoreceptors is reduced by stray light leakage and how this affects *Drosophila* in its natural visual environment.

It is important to note that the broadened spectra we observe in *Drosophila* photoreceptors compared to intracellular recordings, is also due in part to the method of stimulation (wide-field) and recording (ERG), compared to on-axis point-source illumination and intracellular recordings of single cells. Additionally, ERG recordings are from the sum of the stimulated photoreceptors in the eye. Thus, with widefield illumination, as light intensity is increased, additional photoreceptors are stimulated (photoreceptor recruitment). It is therefore more difficult to reach photoreceptor saturation with ERG recordings. Consequently, photoreceptors are tested at a higher light intensity than with point-source illumination and intracellular recordings, which will likely cause broadening of spectral curves. Wide-field illumination, as is experienced in the natural environment, more closely resembles natural illumination than on-axis point source illumination. We suggest that in the case of other red-eye flies, we may see similar shifts in sensitivity of long-wavelength photoreceptors when stimulating with wide-field illumination.

Like other large dipterans, *Drosophila* has a blue absorbing carotenoid present in the distal R7y retinula cells^5^. In *Musca* and *Calliphora* this serves to reduce the light absorbed by the blue-sensitive visual pigment in R7y, instead conferring sensitivity to the UV by the presence of a UV-sensitising pigment^25,26^. This interesting and complex system is not found in *Drosophila*, rather UV-sensitivity is achieved simply by the presence of a dedicated UV-sensitive visual pigment Rh4^3^. However, the presence of this carotenoid pigment in *Drosophila* was not previously understood and its effect on R8y (Rh6) has not been tested until now. When carotenoids were removed from the diet of flies with rescued R8y activity, sensitivity was restored close to the peak of the visual pigment (510 nm) but some effect of light leakage remained at 600 nm. This suggests that the blue-absorbing filter reduces light available to Rh6 at its peak sensitivity (508 nm) and instead extends sensitivity towards the red. These findings could not be replicated by simulating the differences in experimental conditions, either reducing or increasing testing intensity. One generation was sufficient to remove the contribution of the Rh1 UV-sensitising pigment and further generations of carotenoid deprivation did not change the response curve of Rh6 flies (Supplementary Figure S2), indicating that carotenoid filters had been fully removed by one generation.

Carotenoid deprivation also affects the Rh4 rescue flies, which have active R7y photoreceptors that contain the yellow carotenoid filter. When removed, sensitivity is increased at wavelengths greater than 400 nm, suggesting that this filter narrows sensitivity in the UV, potentially increasing wavelength discrimination in that region of the spectrum. We suggest that this filter both contributes to refining the sensitivity of the UV-sensitive R7y (Rh4) photoreceptor and bathochromically shifts the sensitivity of R8y cells by depleting wavelengths of light between 400 and 540 nm in the R7y receptor. In large flies, the yellow filter only shifts R8y sensitivity from 520 to 540 nm but it is not known whether the filter in *Drosophila* absorbs at longer wavelengths, which could in part explain the larger shift we observe. Unfortunately the absorption properties of this carotenoid filter are currently only available for *Musca and Calliphora*^5,20,27^. Interestingly, the overall sensitivity of the UV-sensitive Rh3 and Rh4 rescue flies is relatively unchanged with carotenoid deprivation, whereas the maximum response of Rh5 and Rh6 flies is greatly reduced (Supplementary Figure S1). Chromophore, a derivative of carotenoids, is reduced in flies with carotenoid deprivation, which consequently affects the maturation of opsin protein^28^. While studies have shown that carotenoid deprivation also inhibits opsin expression using some carotenoid-absent food sources (e.g. Sang’s medium), opsin expression is still present in flies raised on yeast-glucose food^29^, which we used here. The apparent lack of reduction in sensitivity of the UV-sensitive Rh3 and Rh4 rescue flies may be explained by the simultaneous reduction in UV-sensitising pigment associated with Rh1, which strongly absorbs UV wavelengths in the outer receptors. This relative increase in available UV light may help to recover sensitivity in Rh3 and Rh4 rescue flies when stimulated with wide-field stimuli. We would not expect to see a similar recovery in Rh5 and Rh6 rescue flies, which are relatively less sensitive to UV-light.

Our findings suggest that at the level of the photoreceptor soma there is no detectable interaction between inner R7/R8 receptor pairs, or inhibition, which has been detected further downstream in the visual pathway at the first visual synapse in the medulla and via the Dm9 pathway^1,2^. This strongly suggests that there is no direct enhancement or inhibition of signal, which could be achieved by gap junctions between neighbouring photoreceptors or by local electrical fields in the surrounding extracellular space. Furthermore, although spectral inhibition occurs between photoreceptor terminals in the medulla^1,2^ it is not detectable upstream in *Drosophila*. If such inhibitory processes typically originate at the terminals and further downstream in other insects, then this may explain why so few studies have described spectral inhibition using intracellular recordings alone.

Our results suggest there may be interactions between outer and inner receptors, indicative of a feedback pathway in the lamina, where both inner and outer receptors interact. Cross-modulation via gap junctions in the lamina is known to occur between the Rh1-mediated motion pathway and R7/R8 colour pathway^30,31^ and was proposed as a mechanism to improve motion discrimination^17^. We suggest that our findings provide some evidence to support this circuit model but they are not strongly conclusive as only subtle changes between spectral shape was observed in double opsin rescues, compared to the expected sum of the single rescue responses. Results may also be affected by the additional complexity of outer receptor ERG responses, due to the feedback from interneurons (large monopolar cells), which modulate photoreceptor response^32^. Recent work has explored spectral interactions between inner and outer photoreceptors by directly recording the activity of photoreceptor axons and inferring photoreceptor response by means of genetic dissection^2^. In these experiments, outer receptors were not shown to significantly contribute to spectral tuning of inner receptors, rather modulation occurred only between inner receptors. Therefore, while we are confident our results do not support direct opponency between inner receptor pairs, we are unable to draw strong conclusions from inner and outer receptor interactions. The addition of responses from all single rescue genotypes is in good agreement with wild type responses, demonstrating that overall, the voltage output of the photoreceptors sum linearly at the level of the retina (Supplementary Figure S5).

For the full characterisation of visual systems, our experiments show the importance of measurements that take into account the modulating effects of screening from distal receptors and ocular pigments. We found that the response of R8y receptor is strongly bathochromically shifted by both the presence of screening pigment and the blue-absorbing yellow filter. The latter also plays a role in refining the sensitivity of the Rh4 UV-sensitive visual pigment, which would likely enhance spectral discrimination. These findings contribute to the greater understanding of the *Drosophila* visual system and will assist in guiding future visual experiments and visual system modelling for which it is vital that the underlying photoreceptor sensitivity is known.

## Methods

### Animals

All *Drosophila melanogaster* stocks were maintained on 12/12 h light/dark light cycle at 22 °C. Flies were reared on either yellow cornmeal or yeast-glucose food, for tests of carotenoid deprivation. Photoreceptor activity was selectively recovered by expression of phospholipase C (PLC) under opsin promotors against a *norpA* background*,* generating single opsin rescue flies. Opsin rescue flies were generated with wild-type screening pigment (red-eye), *w[+] norpA[36]; Rh-norpA* and reduced screening pigment (orange eye), by incorporation of the *mini-white* gene (noted by *w[*+*mC])*, *w[−] norpA[36]; P{w[*+*mC], Rh-norpA}*. All red-eye single opsin rescue flies were generated in a previous study (Rh3: *w[−] norpA[*36*]; P{w[*+*mC], Rh3-norpA[1]},* Rh4: *w[−] norpA[36]; P{w[*+*mC], Rh4-norpA[12]},* Rh5: *w[−] norpA[36]; P{w[*+*mC], Rh5-norpA[20pa]},* Rh6: *w[−] norpA [36]; P{w[*+*mC], Rh6-norpA[5a1]}*^17^). Single rescue flies were crossed to generate double opsin rescue genotypes.

We generated flies with ectopic expression of Rh6 opsin in the outer receptors, under the control of the Rh1 (*ninaE*) promotor, in a white eye mutant background, *w[−] norpA [36]; PBac{actin88F* > *RFP, ninaE* > *norpA}*, *PBac{actin88F* > *GFP, ninaE* > *Rh6}; ninaE *[8]. In order to ectopically express Rh6 in outer photoreceptors of *w[−] norpA *[36] mutant flies, we generated the following two constructs: *pigActGFP-* ninaE > *Rh6* and *pigActmCherry-* ninaE > *norpA*. In all the cloning steps, the insert DNA was amplified by PCR using proper primer combinations (Supplementary Table S1) and integrated into the backbone vector *pigAct88F-GFP* through recombinational cloning (In-Fusion, Takara). Unless otherwise specified, *D. melanogaster w*^*1118*^ genomic DNA was used as a template in the PCR reactions. The *pigAct88F-GFP* vector stems from the *piggyBac* transposon-based plasmid *pigA3GFP*^33^ through replacement of the *3xP3* synthetic promoter present in *pigA3GFP* by the promoter and 5′UTR of *D. melanogaster Actin88F* gene (nucleotides − 1410 to + 647). The Actin88F promoter is active in the thoracic muscles^34^ and thus Actin88F-GFP can be used as a transgenesis marker without interfering with ERG experiments. The Rh1 promoter and 5′UTR (nucleotides − 2788 to + 176; amplified by PCR using genomic DNA isolated from Bloomington stock #52276), the Rh6 coding sequence (including internal introns) and the *SV40* polyadenylation signal (amplified by PCR from the *pigA3GFP* vector) were subsequently inserted into *pigAct88F-GFP* vector in a stepwise manner, giving rise to the final construct *pigActGFP-* ninaE > *Rh6*. For the generation of the *pigActmCherry-* ninaE > *norpA* construct, the *GFP* gene present in *pigAct88F-GFP* was replaced by *mCherry* (amplified by PCR from the vector *pFPV-mCherry*. Addgene #20956) to generate the vector *pigAct88F-mCherry*. This vector was used as a backbone for the cloning of the Rh1 Promoter > *norpA* cDNA > ninaE 3′UTR cassette (amplified by PCR using genomic DNA isolated from the *D. melanogaster* Bloomington stock #52276), giving rise to the final construct *pigActmCherry-* ninaE > *norpA*. Both *pigActGFP-* ninaE > *Rh6* and *pigActmCherry-* ninaE > *norpA* were injected into *Drosophila w*^*1118*^ embryos following standard *piggyBac* transformation protocols. Oregon R–C flies (BDSC_5, Bloomington Stock Centre) were used to test wild-type response and a *norpA* mutant with no rescue was used as a negative control, *w[-] norpA;*+*;*+ (Supplementary Figure S7). All fly lines used in this study are summarised in Supplementary Table S2).

### Light stimulus

Light from a 150 W xenon arc lamp (Cairn Research) was coupled to a monochromator (Cairn Research) with either 1200 or 2400 line-ruled diffraction grating. Long-pass filters were placed in the light path to filter optical harmonics produced by the 2400 grating (< 250 nm, WG280, Schott) and 1200 grating (< 400 nm, GG435, Schott). Intensity of the light was controlled by altering the width of the input and exit slits of the monochromator. Peak wavelength was controlled by the grating angle, yielding a testing range between 315 and 550 nm or 450 and 700 nm in 5 nm steps for the 2400 and 1200 gratings, respectively. Wavelength and photon flux were calibrated at the point of the fly. All spectral measurements were made using spectrophotometers (Avantes AvaSpec 2048 Single Channel spectrometer and Ocean FX, OceanOptics) calibrated to a known light source for measurements of irradiance (DH2000 and DH-3P-BAL-CAL, OceanOptics). Spectral acquisition was controlled using custom MATLAB scripts (v2018a, Mathworks) and conversions to irradiance were carried out according to the manufacturer’s instructions. Irradiance in µW cm^−2^ was converted to photon flux in photons cm^−2^ s^−1^ according to the equation:

$$Photon\,flux=\frac{I\cdot \lambda \cdot {10}^{-9}}{h\cdot c}$$

where *I* is irradiance, *h* is Planck’s constant, *c* is the speed of light and $\lambda$ is wavelength (nm). A measure of total photon flux was calculated by integrating underneath the spectrum curve, which was used for all calibrations and unit conversions were confirmed using OceanView software (OceanOptics). To ensure isoquantal stimuli at each desired wavelength and intensity, we ran an automated calibration protocol using custom MATLAB scripts (Data Acquisition Toolbox, Mathworks), which simultaneously controlled the spectrophotometer and monochromator. The position of entrance and exit slits of the monochromator and the diffraction grating were incrementally adjusted until the desired peak wavelength and total photon flux was reached. Each stimulus was calibrated to within +/− 0.5 nm peak wavelength, calculated using full width of spectrum at half maximum (FWHM) and to within +/− 0.75% total photon flux (for example spectra and bandwidths (FWHM) see Supplementary Figure S8). All stimuli were then measured with the calibrated values to confirm calibration stability to all spectral test intensities, spaced in steps of 0.15 log units (Supplementary Figure S9). Orange eye Rh1, Rh3 , Rh4 and Rh5 rescue flies were illuminated by a deep red 660 nm 4 die LED (LZ4-00R208, LED Engin) with light passing through three stacked long pass glass absorbance filters (RG630, Schott) within a 25 mm optics tube (Thorlabs) to remove short wavelengths below 600 nm. The peak wavelength was measured at 670–675 nm. It was placed 18 cm from the fly at a slight angle to the test light, which enabled full illumination of the testing eye without being occluded by the ERG apparatus or causing electrical interference. High intensity blue light can cause a build-up of Rh1 metarhodopsin, which is in excess of the available arrestin that blocks the continuation of the phototransduction cascade. This occurs more readily in flies with reduced or absent screening pigment, causing a prolonged depolarizing afterpotential (PDA), but can be reversed by photoconversion of the Rh1 metarhodopsin with addition of long-wavelength light^35^. Preliminary testing indicated that long-wavelength light assisted in the recovery of response in Rh1 and Rh5 rescue flies.

### ERG

Animals between 4 and 12 days after eclosion were anesthetised on ice and immobilised on a metal cone using ultraviolet curing adhesive (Norland Optical Adhesive NOA68). Electroretinogram (ERG) recordings were made using borosilicate micropipettes (ID 0.5 mm, OD 1.0 mm, length 10 cm, Sutter, BF100-50-10) pulled on a Sutter P-2000 laser puller (settings: Heat 350, Fil 4, Vel 50, Del 224, Pul 150). Electrodes were filled with insect saline (103 mM NaCl, 3 mM KCl, 20 mM BES, 10 mM trehalose, 20 mM sodium bicarbonate, 1 mM sodium phosphate monobasic, 2 mM CaCl_2_ and 4 mM MgCl_2_, all Sigma Aldrich) using pipette fillers (Microloader E5242956003, Eppendorf). Recordings were measured from near the equator of the eye with a broken pipette touching the eye (creating a good saline junction) and positioned using a motorized micromanipulator (Sensapex). The reference electrode was inserted slightly inside the median ocellus using a manual manipulator. Light was delivered to the fly via a UV transmissive 5 mm liquid light guide (Lumatec series 300) and silica bi-convex lens with 25.4 mm focal length (Newport SBX019, USA), arranged to maximise light stimulation at the point of the fly. All recordings were made within a Faraday cage and responses were amplified (MultiClamp 700B amplifier, Molecular devices; EXT-02F, NPI or BA-03X, NPI). Both stimuli and data acquisition were controlled using a DAQ board (National Instruments) in conjunction with the software, Ephus^36^.

### Experimental design

To determine the stimulus–response (V-log(I)) function, each animal was tested with a series of 200 ms light pulses every 10 s that increased in intensity over a possible range of 6 log units. Red eye flies were tested between 1.14 × 10^12^ and 3.60 × 10^16^ photons cm^−2^ s^−1^ (1200 grating) and between 1.14 × 10^11^ and 3.60 × 10^16^ photons cm^−2 ^s^−1^ (2400 grating). Orange eye flies were tested between 3.60 × 10^10^ and 6.40 × 10^15^ photons cm^−2^ s^−1^. Each intensity was repeated 10 times followed by a pause of 100 s. The wavelength each V-log(I) test was chosen according to previous estimates of peak sensitivity for the test visual pigment: Rh1, 485 nm; Rh3, 345 nm; Rh4, 370 nm; Rh5, 440 nm; Rh6 540 nm^4^. Peak wavelength for Rh6 was adjusted after preliminary tests indicated longer-wavelength peak sensitivity. The intensity at half maximum response was calculated from the V-log(I) curve and the closest isoquantal test intensity was used for spectral tests. In cases where no obvious photoreceptor saturation had occurred, this value was estimated from the fitted curve.

To test spectral response, animals were stimulated with isoquantal flashes every 5 s at all test wavelengths with randomised presentation. Test wavelengths were divided into three blocks from lowest to highest wavelength and randomised within. Stimuli were always presented from these categories in order from low to high, to ensure a balanced order of testing across the wavelength range. Each wavelength was tested 10 times concurrently and the last 5 responses were used for analysis. All genotypes were tested with wavelengths of 315–550 nm and those with long wavelength responses (e.g. Rh1 and Rh6) were also tested with 450–700 nm, in steps of 5 nm. All animals were dark adapted for 30 min prior to the V-log(I) test and subsequently a further 15 min before each spectral sensitivity test.

### Analysis

The responses from 6 animals were used for each experiment with the exception of the Rh6 rescue flies raised for two generations on yeast-glucose food where 4 animals were used for spectral testing. ERG responses were normalized to a zero baseline using the average of 100 ms prior to stimulus onset. Photoreceptor response was calculated as the change in voltage between the zero baseline and minimum voltage during the 10 ms before the end of the light flash for spectral tests and 20 ms for V-log(I) tests. The last 5 photoreceptor responses from each set of 10 repeats was used for V-log(I) and spectral sensitivity tests. These responses were averaged and mean responses used to compare genotypes. Animals with low or noisy ERG responses indicating a poor-quality preparation or inadequate electrode connection were not used for further analysis. V-log(I) data were fitted to the Naka–Rushton function:

$$\frac{V}{{V}_{max}}= \frac{{I}^{n}}{{I}^{n}+ {K}^{n}}$$

where *V* is the photoreceptor response, *V*_*max*_ is the maximum response, I is the light intensity and *K* is the light intensity required to achieve half of *V*_*max*_^37^ and *n* is the slope. The intensity at half maximum response (*K*) was used for spectral tests to ensure all testing was carried out in the linear portion of the V-log(I) curve, where possible. Where data did not fit well to a Naka–Rushton function, *K* was estimated from a fitted sigmoid function. Due to the low response of carotenoid-deprived Rh5 and Rh6 rescue flies, these were tested at the maximum possible intensities Rh5: 7.18 × 10^15^ photons cm^−2^ s^−1^ and Rh6: 1.02 × 10^16^ photons cm^−2^ s^−1^). For spectral sensitivity tests, outliers greater than three scaled median absolute deviations from the median of the 5 responses were first removed then the mean was taken. Data were smoothed using a Savitzky-Golay filter (data window 15 nm) then each spectral curve was normalized per individual and averaged across all individuals in each experiment.

To combine spectral sensitivity curves from tests using both the lower and higher wavelength gratings all non-normalized curves were joined at the 450–550 nm overlap region and an average fit was derived from the fit of all 21 points. The responses for Rh1 opsin rescue flies were larger in the 450–700 nm range than those in the 315–550 nm range likely a result of increased metarhodopsin stimulation at these longer wavelengths. Thus, to better estimate the long-wavelength tail, a scale factor of 0.5777 was calculated at the mean of both curves at its peak (490 nm) and applied to the longer wavelength spectral curve before combining. The joined curves were then normalized between 0 and 1 and analysed as outlined previously. For the 450–550 nm region of cross over between both curves, means and standard deviations were calculated for all 12 points. For a comparison of photoreceptor pair responses, the mean of non-normalized sensitivity curves from single rescue flies were summed pair-wise according to the order of light stimulus and compared with the response curves of double rescue flies. All analyses were performed in MATLAB (v2018a and v2018b, Mathworks), using custom scripts. Visual pigment templates were generated using R package PAVO^38^. Two sample Student’s t-tests were carried out with Bonferroni correction for multiple sampling between sensitivity curves with the exception of comparisons made between double opsin rescues tested at different light intensities, which were tested using paired t-tests. All statistical tests were carried out in MATLAB (v2018b, Mathworks).

## Supplementary information

Supplementary Information.

Supplementary Data 1.

Supplementary Data 2.
