# Supplementary material for: The spectral sensitivity of Drosophila photoreceptors
Source: Sci Rep. 2020 Oct 26;10:18242. doi: 10.1038/s41598-020-74742-1 (PMC7588446; doi:10.1038/s41598-020-74742-1)
Supplement: Supplementary file 1 — Supplementary Information. [file 41598_2020_74742_MOESM1_ESM.pdf]

The spectral sensitivity of *Drosophila* photoreceptors

Camilla R. Sharkey[1*], Jorge Blanco[1], Maya M. Leibowitz[2], Daniel Pinto-Benito[2], Trevor J. Wardill[12*]

[1] Department of Ecology, Evolution and Behavior, University of Minnesota, Saint Paul, MN 55108, US
[2] Department of Physiology, Development and Neuroscience, University of Cambridge, Cambridge, CB2 3EG, UK

*Correspondence: camilla.r.sharkey@gmail.com & twardill@umn.edu

Supplementary Data

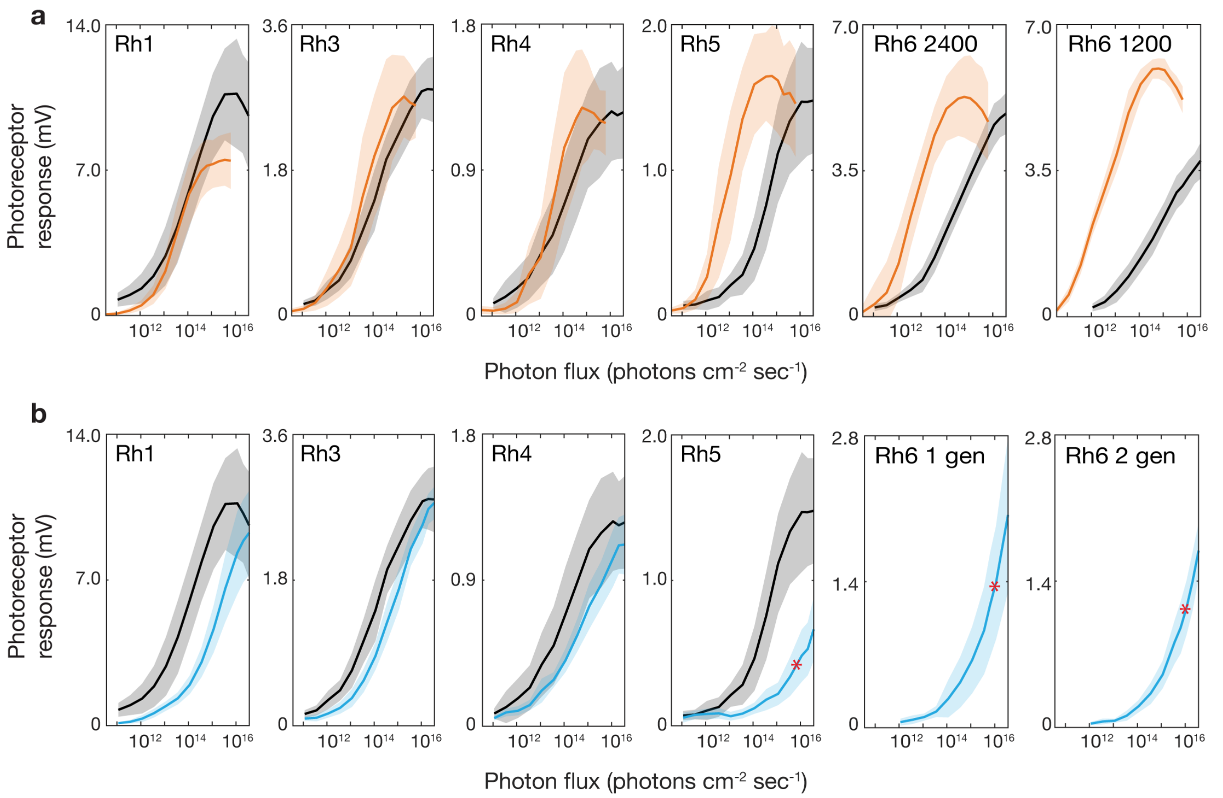

Figure S1. (a) Stimulus-response curves (V-log(I)) from single opsin rescue flies (Rh1, Rh3 - 6) with red (black lines) or low levels of screening pigment (orange lines), tested over 6 log units of light. Photon flux is on a logarithmic scale. Rh6 V-log(I) responses using the 2400 line (315 - 550 nm) and 1200 line (450 - 700 nm) grating are shown. Rh1, Rh3, Rh4 and Rh5 rescue flies with reduced screening pigment were also illuminated with deep red background illumination. (b) Stimulus-response curves for red eye single opsin rescue flies (Rh1, Rh3, Rh4 and Rh5) raised on cornmeal (black) and carotenoid-deprived single opsin rescue flies (blue). Flies raised for one generation on yeast glucose fly food (Rh3, Rh4, Rh5, Rh6 1 gen) and two generations (Rh1 and Rh6 2 gen) are shown. Rh6 deprived flies were tested with the 1200 line grating. The red asterisks indicate the intensities at which carotenoid-deprived Rh5 and Rh6 spectral sensitivity tests were carried out (Rh5: 7.18×10^{15} photons cm^{-2} sec^{-1} and Rh6: 1.02×10^{16} photons cm^{-2} sec^{-1}), which are the maximum intensities of isoluminant stimuli that can be generated with this system for each grating. Note, there is a scale change between (a) and (b) for Rh6 rescue fly responses. All error shown is standard deviation and n = 6 for all V-log(I) curves with the exception of carotenoid-deprived Rh6 2 gen, which is n = 4.

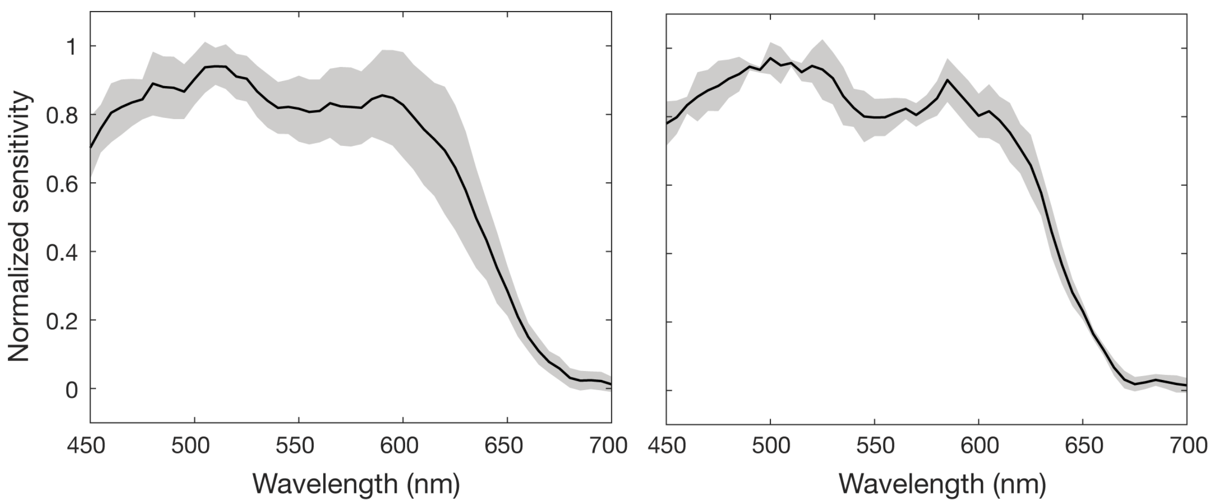

Figure S2. Spectral response of red-eye Rh6 rescue flies with one (left) or two (right) generations of carotenoid deprivation tested at 1.02 x10^{16} photons cm^{-2} s^{-1}. Sample sizes are n = 6 and n = 4, respectively. Error shown is standard deviation.

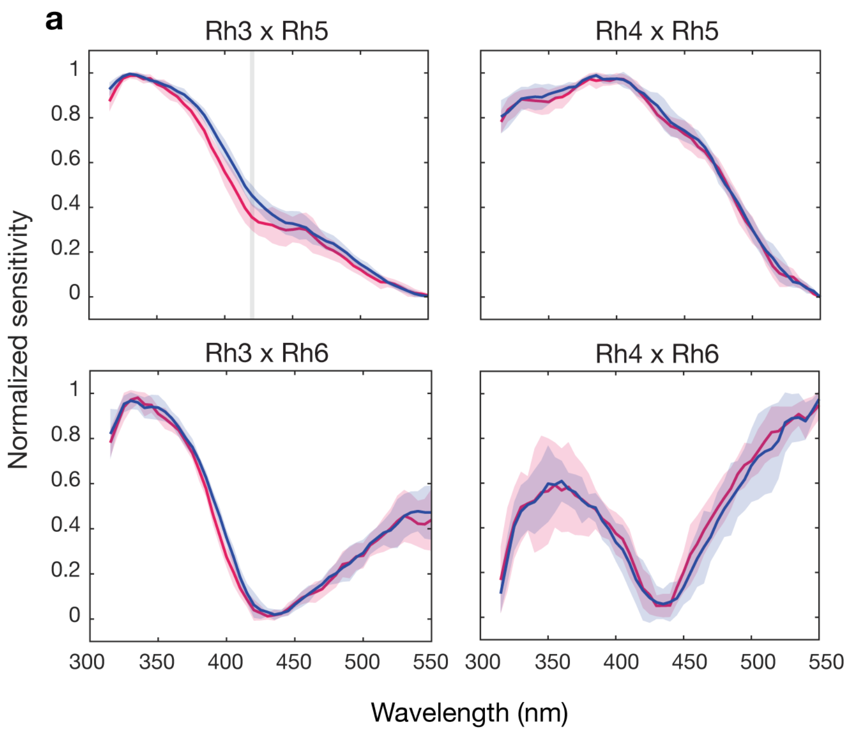

Figure S3. (a) Normalized spectral sensitivity curves of double opsin rescues with two inner photoreceptor types active. Double opsin rescue flies were tested at an intensity derived from the V-log(I) test at either the Rh3/Rh4 peak sensitivity (pink) or Rh5/Rh6 peak sensitivity (blue). A significant change in shape was only observed in Rh3 x Rh5 flies at 420 nm (paired Student's t-test, $t(5) = -8.908$, $P<0.001$). (b) Normalized spectral sensitivity curves of double opsin rescues with one inner photoreceptor type and outer receptors active. Flies were tested at an intensity derived from the V-log(I) test at either the Rh1 peak sensitivity (pink) or at the peak sensitivity of the inner receptor opsin Rh3 - Rh6 (blue). Shading denotes significance between sensitivity curves using a paired Student's t-test at $p \leq 0.001$. Error shown is standard deviation. n = 6 for each double rescue and flies were tested twice, once at each intensity. In three cases where the same intensity was chosen from each V-log(I) test, a repeat spectral test was carried out.

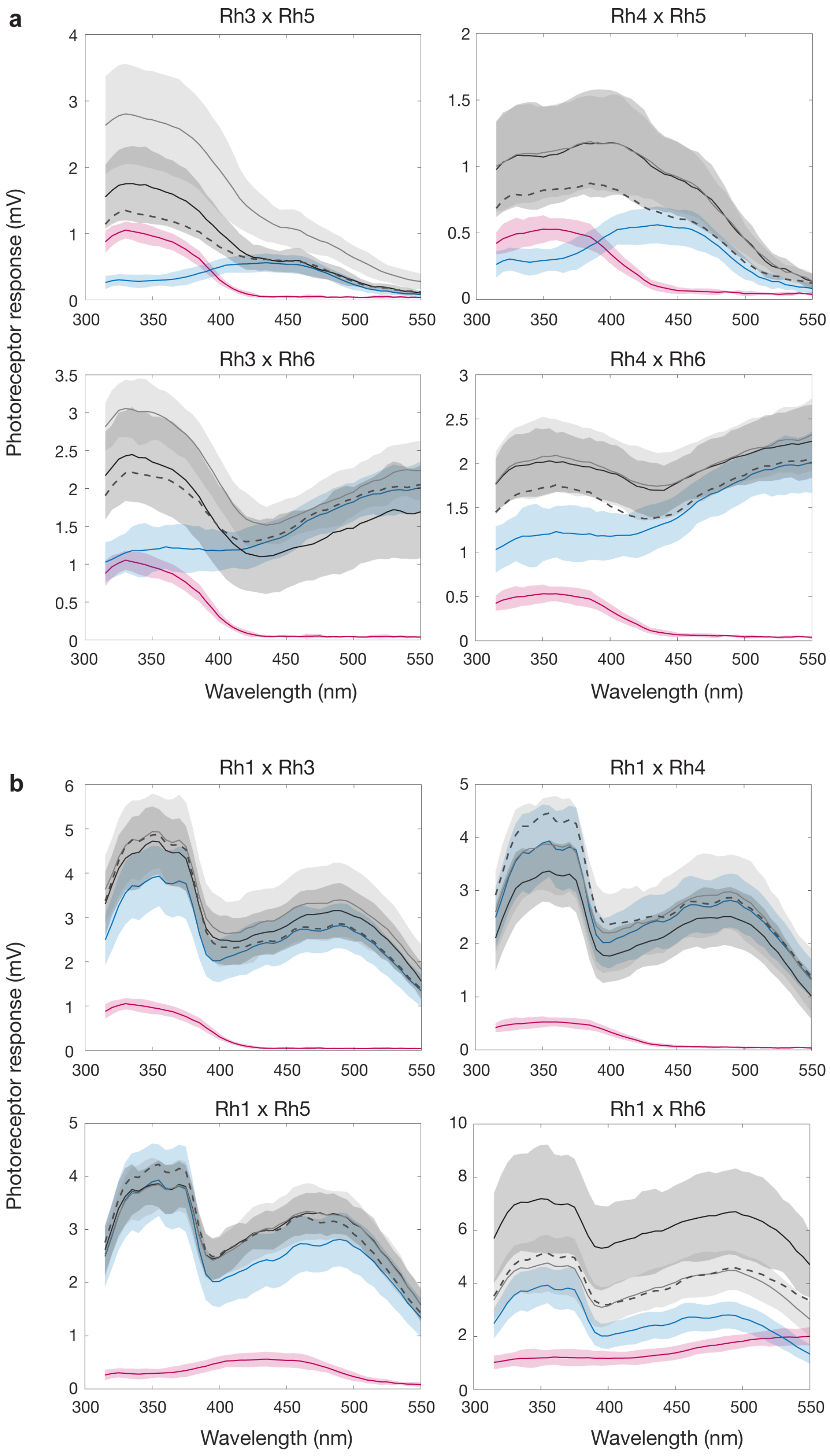

Figure S4. (a) Voltage responses of single (pink: Rh3 or Rh4 and blue: Rh5 or Rh6) and double opsin rescue flies tested at an intensity derived from the V-log(I) response of either Rh3/Rh4 (dark grey) or Rh5/Rh6 (light grey). The algebraic sum of the single responses (dashed lines). (b) Voltage responses of single (pink: Rh3 - Rh6 or blue: Rh1) and double opsin rescue flies tested at an intensity derived from the V-log(I) response of either Rh3 - Rh6 (dark grey) or Rh1 (light grey). Error shown is standard deviation. n = 6 for all single and double opsin rescues. For each double opsin rescue, six flies were tested twice, at both intensities.

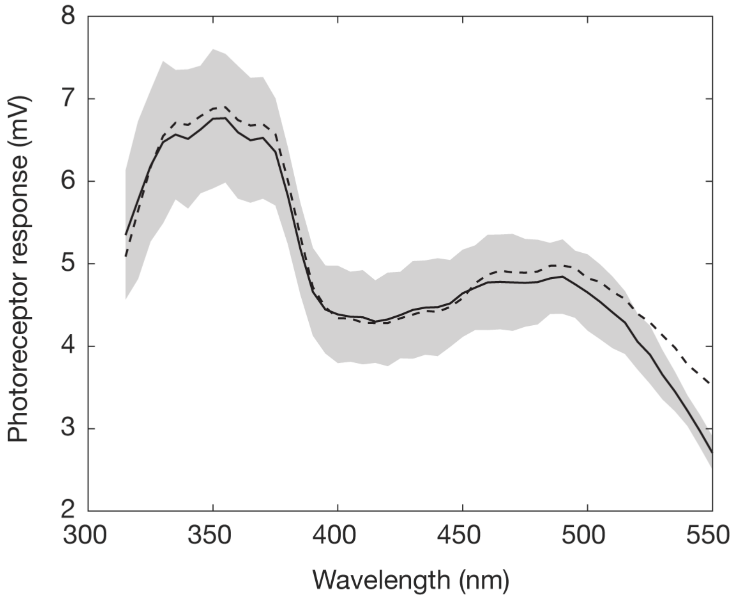

Figure S5. Photoreceptor response of wild-type flies (solid line) and the algebraic sum of mean responses from all red-eye single opsin rescues (dashed line). Error shown is standard deviation and n = 6 for wild-type flies.

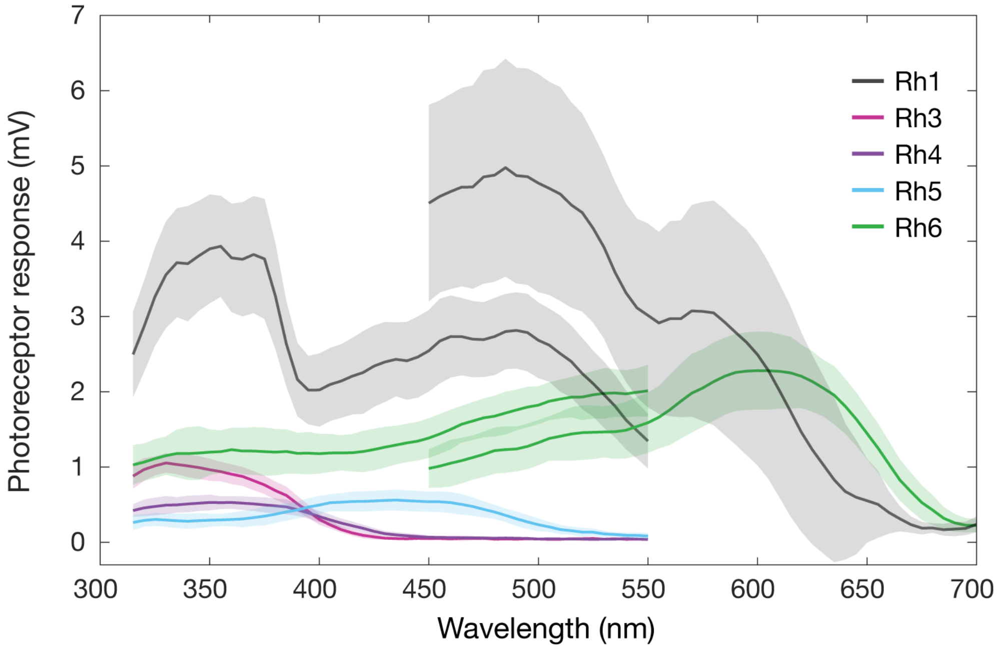

Figure S6. Photoreceptor responses of each red-eye opsin rescue fly (n = 6). Rh1 and Rh6 rescue flies were tested in the range of 315 to 550 nm and also 450 to 700 nm to span the full range of sensitivity (n = 6 for each Rh1 and Rh6 sensitivity curve). Error shown is standard deviation. For data values see Supplementary Data.

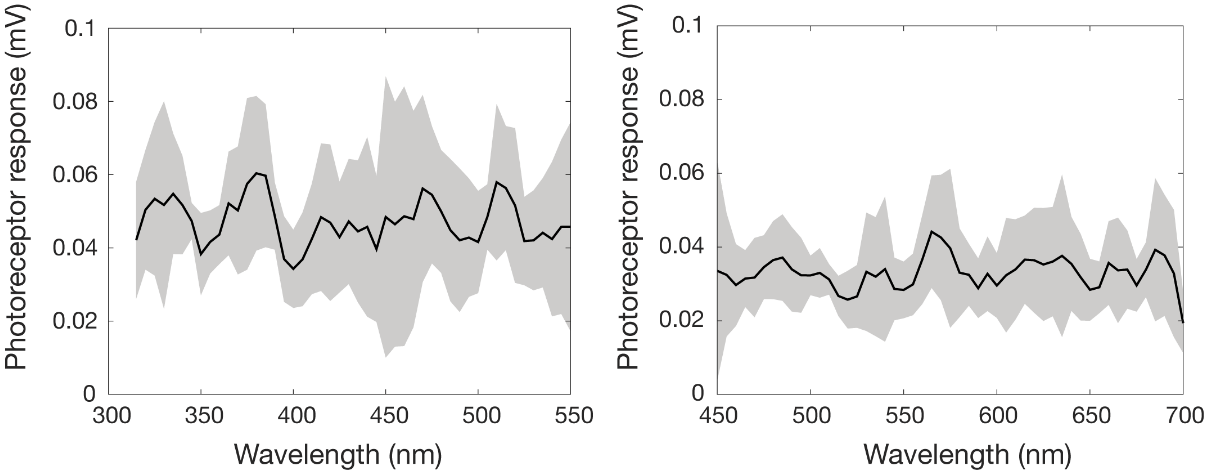

Figure S7. Photoreceptor response of control flies *w[-] norpA;+;+* indicating the baseline response from flies with no photoreceptor response. Control flies were tested across the test wavelength ranges: 315 - 550 nm and 450 - 700 nm. Flies were tested at 7.18 x 10^{15} and 3.60 x 10^{15} photons cm^{-2} s^{-1}, respectively. n = 6 for each test wavelength range. Error shown is standard deviation.

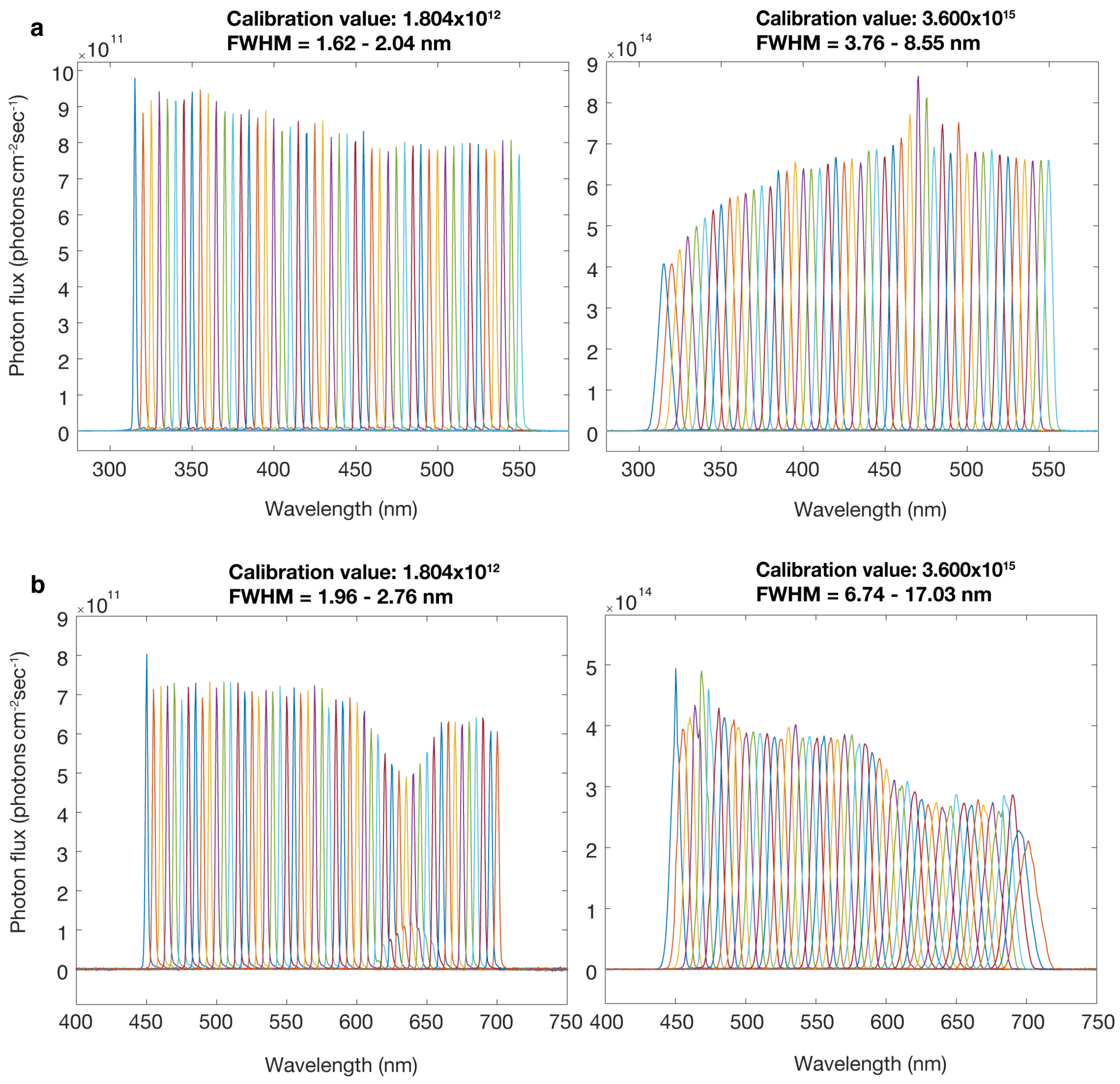

Figure S8. Example spectra of isoquantal stimuli calibrated to low (1.804 x 10^{12}) and high (3.6 x 10^{15}) target calibration values of total photon flux (area under the spectrum). Each spectrum represents one calibration point, in 5 nm steps from 315 - 550 nm (a) and 450 - 700 nm (b). Bandwidth ranges across all wavelengths were calculated using the full width of spectrum at half maximum (FWHM).

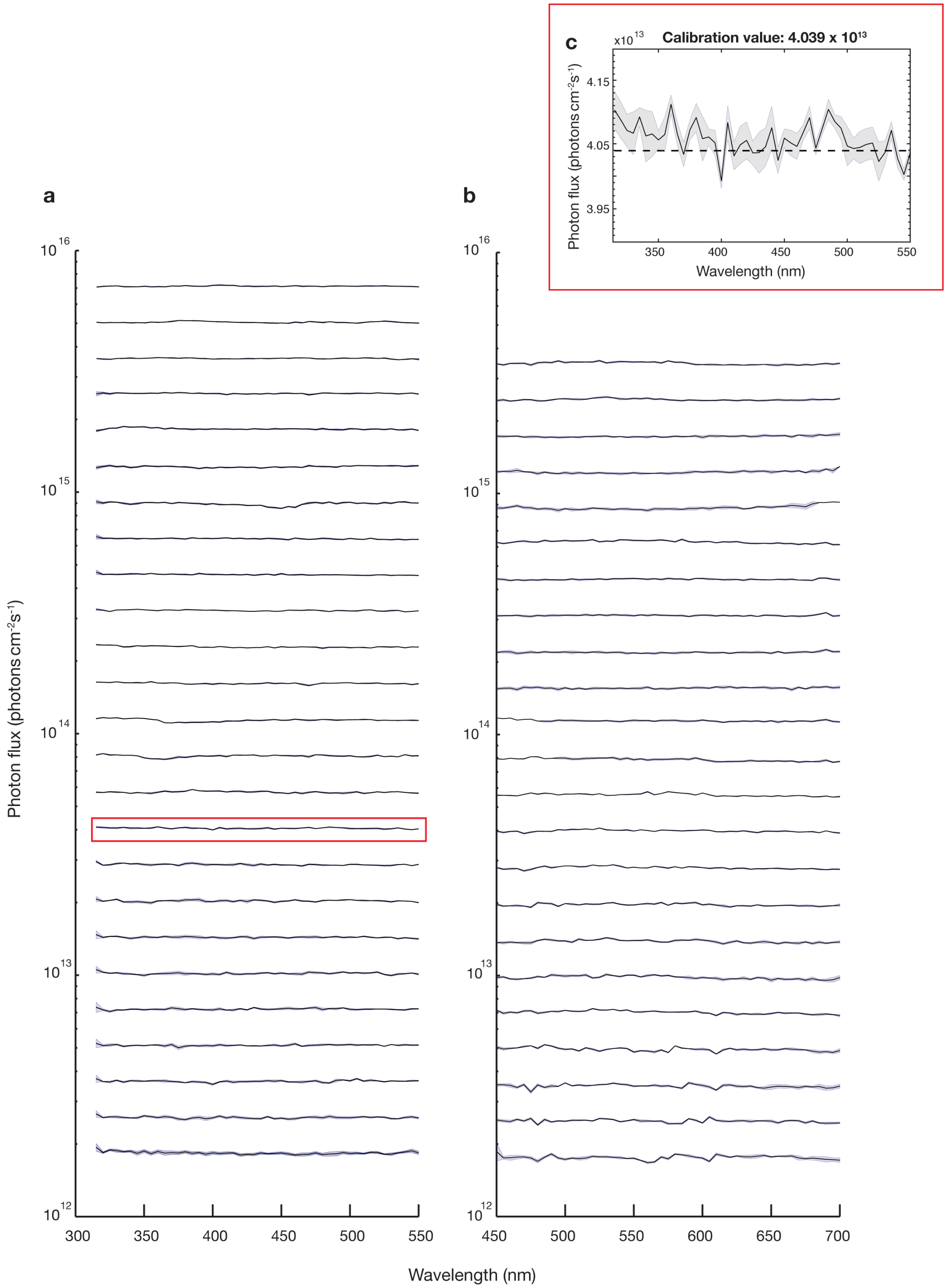

Figure S9. Example of measurements made after calibration to spectral test intensities in steps of 0.15 log units and wavelengths for (a) the 2400 and (b) 1200 line ruled diffraction grating. The y axis is on a log scale. Five measurements were taken per calibration point and the average taken. Magnified version of indicated calibration measurements (c) with calibration reference value shown (dashed line). Error shown is standard deviation.

Primer	Sequence
Act88F-F	TTTGCGGTTTACCGGATGCACAATAGGCAAATTTAGTT
Act88F-R	CATGGTGGCGACCGGCTTGGCAGTTGTTTATCTGGAA
Rh1-F	ACGCGTACGGCGCGCCTTACATACATACTAGAATTC
Rh1-R	CGCGGCCGCTCCTAGGTGTGTTTTGGTTACTGGCTG
Rh6-F	CCAAAACACACCTAGGCCACCATGGCCAGCCTGCATCCCCC
Rh6-R	CGCGGCCGCTCCTAGGCTAAGCCTGCGATTCGCTAA
SV40-F	AGCGAAGTAACCTAGGAGCGGCCGCGACTCTAGATC
SV40-R	TTGGATCCATATATAGGGCCCTAAGATACATTGATGAGTTTGGAC
mCherry-F	CCGGTCGCCACCATGGTGAGCAAGGGCGAGGAGGAT
mCherry-R	CGCGGCCGCTCCTAGGCTACTTGTACAGCTCGTCCA
Rh1>norpA-F	CATCAATGTATCTTAGGGCCCTTACATACATACTAGAATTC
Rh1>norpA-R	TTGGATCCATATATAGGGCCCAGCTTGGGCTGCAGGTCGAC

Table S1. Primers used for the PCR amplification of the insert DNAs to generate *w- norpA[36]* mutant flies ectopically expressing Rh6 in the outer photoreceptors. The sequences for recombination are underlined.

Description	Genotype	Stock/ID number	Source
Wild type	Oregon R-C	BDSC_5	Boomington Stock Centre, IN
Red eye Rh1 opsin rescue	norpA[36].CS; P{w[+mC]=ninaE>norpA.W}2	-	This study. X from red eye Rh3 opsin rescue, 1st from Bloomington Stock Centre (BDSC_52276)
Red eye Rh3 opsin rescue	norpA[36].CS; P{w[+mC]=Rh3>norpA[1]}	-	(Wardill *et al*. 2012)
Red eye Rh4 opsin rescue	norpA[36].CS; P{w[+mC]=Rh4>norpA[12]}	-	(Wardill *et al*. 2012)
Red eye Rh5 opsin rescue	norpA[36].CS; P{w[+mC]=Rh5>norpA[20pa]}	-	(Wardill *et al*. 2012)
Red eye Rh6 opsin rescue	norpA[36].CS; P{w[+mC]=Rh6>norpA[5a1]}	-	(Wardill *et al*. 2012)
Orange eye Rh1 opsin rescue	w[-] norpA[36]; P{w[+mC]=ninaE>norpA.W}2	BDSC_52276	Boomington Stock Centre, IN
Orange eye Rh3 opsin rescue	w[-] norpA[36]; P{w[+mC]=Rh3>norpA[1]}	-	This study. X from norpA control, 1st from Wardill *et al*. (2012)
Orange eye Rh4 opsin rescue	w[-] norpA[36]; P{w[+mC]=Rh4>norpA[12]}	-	This study. X from norpA control, 1st from Wardill *et al*. (2012)
Orange eye Rh5 opsin rescue	w[-] norpA[36]; P{w[+mC]=Rh5>norpA[20pa]}	-	This study. X from norpA control, 1st from Wardill *et al*. (2012)
Orange eye Rh6 opsin rescue	w[-] norpA[36]; P{w[+mC]=Rh6>norpA[5a1]}	-	This study. X from norpA control, 1st from Wardill *et al*. (2012)
White eye Rh6 opsin ectopically expressed in rescued outer receptors	w[-] norpA[36]; PBac{actin88F>RFP, ninaE>norpA}, PBac{actin88F>GFP, ninaE>Rh6}; ninaE[8]	-	This study. w[-] norpA[36] from Roger Hardie, ninaE[8] from William Pak
White eye norpA control	w[-] norpA[36]; +; +	-	Gift from Roger Hardie (also called P24)

Table S2. *Drosophila melanogaster* genotypes used in this study.
